# Efficacy and Clinical Characteristics of Liraglutide in Japanese Patients With Type 2 Diabetes

**DOI:** 10.14740/jocmr2237w

**Published:** 2015-07-24

**Authors:** Daisuke Ito, Takujiro Iuchi, Susumu Kurihara, Ikuo Inoue, Shigehiro Katayama, Kouichi Inukai

**Affiliations:** a Division of Endocrinology and Diabetes, Saitama Medical University, 38, Morohongo, Moroyama, Iruma-gun, Saitama 350-0495, Japan; b Division of Internal Medicine, Ogawa Red Cross Hospital, 1525, Ogawa, Ogawa, Hiki-gun, Saitama 355-0397, Japan; c Division of Diabetes and Endocrinology, Higashiyamato Hospital, 1-13-12, Nangai, Higashiyamato, Tokyo 207-0014, Japan

**Keywords:** Type 2 diabetes, Liraglutide, GLP-1 receptor agonist, Long-acting, Body weight, Incretin

## Abstract

**Background:**

Liraglutide was first released in Japan as a long-acting once-daily glucagon-like peptide-1 receptor agonist. The maximum dose in Japan is 0.9 mg/day, which is half of that used in the United States and the European Union (1.8 mg/day). The efficacy of this maximum allowable dose of liraglutide for Japanese patients and the profiles of those patients for whom this agent should be recommended remain unclear.

**Methods:**

This study aimed to examine the effective use of liraglutide in Japanese type 2 diabetic patients. We administered liraglutide to 60 patients, who had been managed with oral hypoglycemic agents or diet and exercise therapy only, during a period of 6 months.

**Results:**

Though HbA1c levels significantly decreased, by approximately 1.5%, after 6 months of liraglutide administration, no significant changes in body weights were observed. The 0.6 mg dose was effective in approximately 40% of patients. In contrast, the effects of a dose increase from 0.6 mg to 0.9 mg were small. The greatest efficacy, as shown by a 2.5% HbA1c decrease, was achieved in non-obese patients. Thus, efficacy decreased as the degree of obesity increased. In addition, efficacy was higher in patients who had a diabetes duration of less than 10 years and was also higher in the group that had a low sulfonylurea (SU) index, when we define the SU index as mg/glimepiride × years of treatment.

**Conclusions:**

As appetite suppressions and associated decreases in body weights were not observed in obese patients, the efficacy of liraglutide at 0.9 mg did not appear to be high. Rather, it appeared to be highly effective for patients who were non-obese and for whom amelioration of blood glucose elevations could be anticipated via the stimulation of insulin secretion. Therefore, we found that liraglutide at doses of 0.9 mg was highly effective in non-obese patients who were in the early stages of diabetes and was particularly effective in patients who had not yet been administered SU agents.

## Introduction

Incretins are hormones secreted from the gastrointestinal tract that, in response to food intake, stimulate pancreatic β cells to secrete insulin. To date, two types of incretins have been confirmed: glucose-dependent insulinotropic polypeptide (GIP), which is secreted from K cells in the proximal small intestine, and glucagon-like peptide-1 (GLP-1), which is secreted from L cells in the distal small intestine. As both types of incretins are quickly degraded by dipeptidyl peptidase-4 (DPP-4), their activities are abolished within several minutes. GLP-1 receptor agonists, which cannot be degraded by DPP-4, increase GLP-1 actions, which are likely to be decreased in type 2 diabetic patients [1-3].

Three types of GLP-1 receptor agonists that are currently available in Japan are administered on four dosing schedules: once-daily liraglutide, twice-daily or once-weekly exenatide and once-daily lixisenatide [4-7]. Of these, liraglutide was the first GLP-1 receptor agonist available for use in Japan and was launched on the market in 2010. On the basis of phase II study results, the maximum dose covered by insurance was limited to 0.9 mg/day, which is half of that used in the US and Europe (1.8 mg/day). The global liraglutide effect and action in diabetes (LEAD) studies have demonstrated the efficacy and safety of liraglutide [8-13]. Similarly, two Japanese studies found liraglutide to be both effective and safe for clinical use [14-17]. However, prior studies showed the glucose-lowering effects of incretin-associated agents, i.e. DPP4 inhibitors and GLP-1 receptor agonists, in Japanese populations to be superior to those in westerners and that the decrease in body weight with GLP-1 receptor agonist treatment was small in the Japanese cohorts [18-22]. Although liraglutide has been in clinical use for 5 years, no clear consensus has been reached concerning racial differences in the effects of GLP-1 receptor agonists. Furthermore, the efficacy of the maximum allowable dose of liraglutide for Japanese patients and the profiles of those patients for whom this agent should be recommended remain unclear.

The present study aimed to examine the effective use of liraglutide. We administered liraglutide to 60 patients, who were treated with oral hypoglycemic agents or diet and exercise therapy only. We subsequently examined the efficacy of this agent in various patients after differential analyses according to background factors. We obtained novel findings, anticipated to lead to the appropriate use of liraglutide.

## Materials and Methods

### Subjects

This was a multicenter, open-label, prospective, observational clinical study. After receiving approval from the ethics review board of the Saitama Medical University Hospital, which played a central role in conducting the present study, we enrolled 60 patients, who had been treated with oral hypoglycemic agents or diet and exercise therapy only, in multiple facilities as study participants. Three patients dropped out, because they could not continue to visit the participating hospitals. The patient characteristics are shown in Table 1. Study subjects had HbA1c levels of 7.0% (NGSP) or more and/or were highly prone to obesity (mean body mass index (BMI) of approximately 28 kg/m^2^). In the present study, patients received once-daily subcutaneous injections of liraglutide before breakfast. Doses were started at 0.3 mg and after a 1-month observation period on the 0.6 mg dose, their doses were increased to 0.9 mg, according to patient conditions. The reasons that we did not uniformly increase doses in all patients are as follows: 1) some patients developed gastrointestinal adverse effects at a dose of 0.6 mg; 2) upon observation of the clinical course at a dose at 0.6 mg, it was clear that blood glucose levels were sufficiently controlled in some patients. In terms of this protocol, the present study differs from phase III studies of liraglutide performed in Japan, in which doses were uniformly increased to 0.9 mg. On the basis of the above findings, we believe that our protocol better represents actual clinical conditions. With regard to oral hypoglycemic agents, approximately half of the patients were already receiving treatment with a sulfonylurea (SU) drug. Administration of SU drugs was continued at reduced doses, whereas oral hypoglycemic agents, such as α-glucosidase inhibitors, thiazolidinedione derivatives, and DPP-4 inhibitors, were in principle discontinued.

**Table 1:** Baseline Characteristics

Number of subjects (male/female)	30/27
Age (years)	58.0 ± 12.0
Duration of diabetes (years)	10.6 ± 6.3
Body weight (kg)	72.1 ± 18.6
BMI (kg/m^2^)	27.9 ± 5.9
HbA1c (NGSP) (%)	9.5 ± 1.6
2 h postprandial plasma glucose (mg/dL)	230.2 ± 83.3
2 h postprandial CPR (ng/mL)	3.3 ± 2.0

### Outcome measures

Routine observation items for the presence of adverse events included HbA1c levels, blood glucose levels measured 2 h after breakfast, C-peptide levels, body weight and the incidence of hypoglycemia. Exclusion criteria at the time of enrollment included moderate or severe hepatic dysfunction, renal dysfunction (serum Cr > 2.0 mg/dL) and unstable retinopathy. Written consent was obtained from all patients before study participation.

### Statistical analysis

Data are presented as the mean ± standard error. The probability (P) value was determined using the paired *t*-test and a P value < 0.05 was considered to indicate a statistically significant difference. All analyses were performed using STATASE 11.

## Results

**Figure 1:**
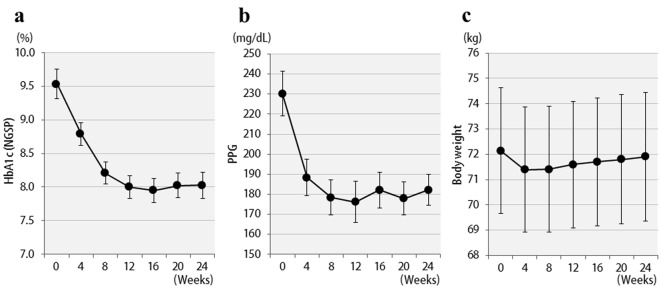
Changes in clinical parameters for 24 weeks after liraglutide treatment in 57 patients: (a) HbA1c (NGSP); (b) 2 h postprandial plasma glucose; (c) body weight.

**Figure 2:**
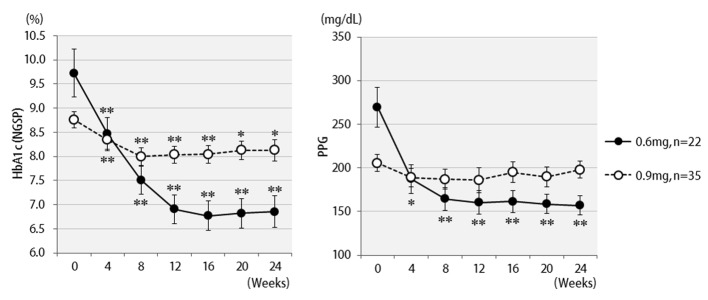
Changes in HbA1c (NGSP), 2 h postprandial plasma glucose for 24 weeks by different liraglutide doses received: 0.6 mg (n = 22) or 0.9 mg (n = 35). **P < 0.001, *P < 0.05 compared with the values at baseline.

Changes in HbA1c concentrations, postprandial blood glucose levels, and BMI values of all 57 patients are shown in Figure 1. Six months after starting liraglutide administration, marked decreases in mean HbA1c and postprandial plasma glucose levels were observed. In contrast, no significant changes in mean body weights were observed though there were temporary decreases in body weights at the time of initial liraglutide administration. The changes in final doses are shown in Figure 2. At the end of this study, 22 patients were still being treated with 0.6 mg, and 35 patients with 0.9 mg of liraglutide. It should be noted that the 0.6 mg dose was effective in approximately 40% of patients overall. While a marked decrease (3%) in HbA1c levels was observed in patients who received the 0.6 mg dose, only a small decrease (< 1% decrease in HbA1c levels) was observed in the group that received the higher 0.9 mg dose. Therefore, the 0.6 mg dose appeared to be sufficient in most Japanese patients for whom liraglutide was effective. However, increasing the dose to 0.9 mg did not necessarily lead to greater HbA1c reductions. Thus, from the viewpoint of cost effectiveness, it is recommended to first observe the courses of patients given the 0.6 mg dose and then increase the dose to 0.9 mg as required.

**Figure 3:**
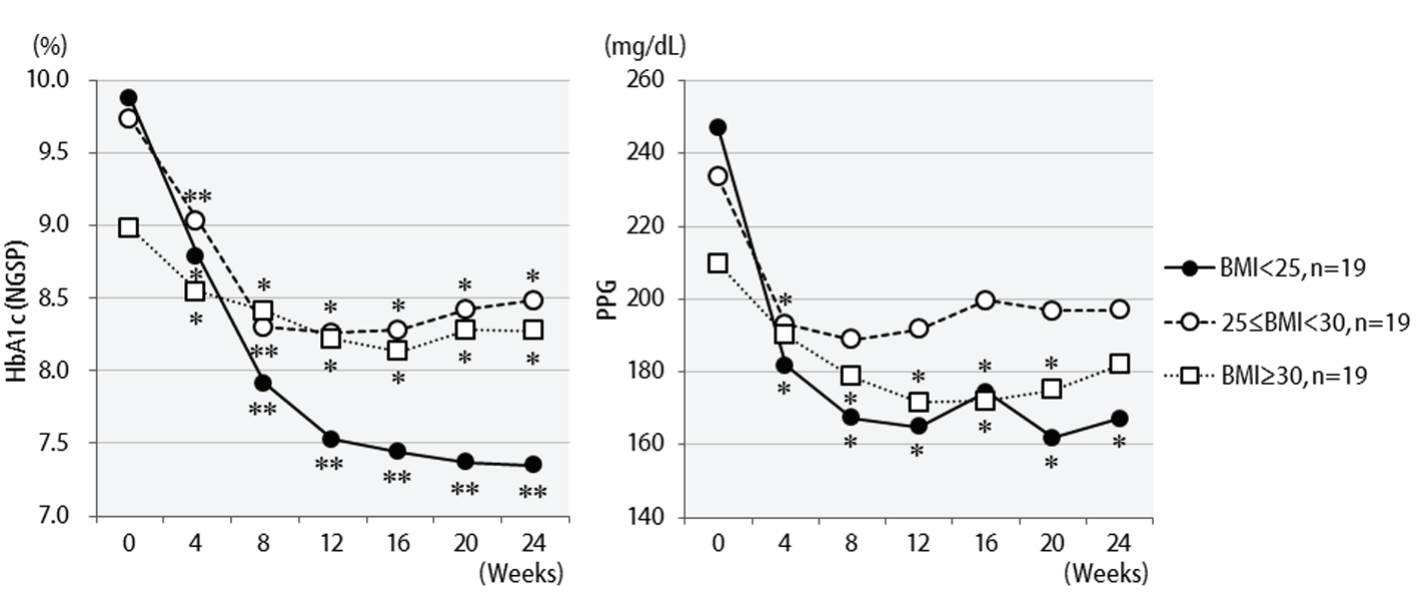
Changes in HbA1c (NGSP), 2 h postprandial plasma glucose for 24 weeks after liraglutide introduction by body mass index difference: less than 25 (n = 19), 25 to 29 (n = 19), or over 30 (n = 19). **P < 0.001, *P < 0.05 compared with the values at baseline.

**Figure 4:**
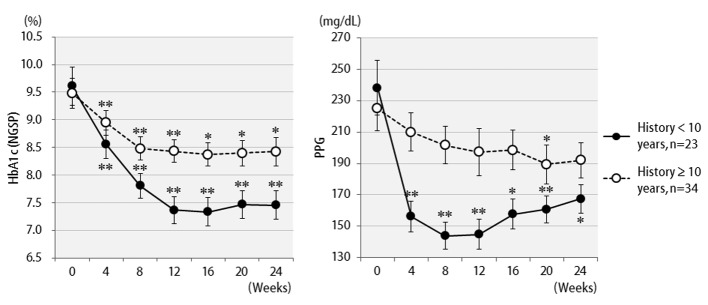
Changes in HbA1c (NGSP), 2 h postprandial plasma glucose for 24 weeks after liraglutide introduction by difference in duration of diabetes: less than 10 years (n = 23) or over 10 years (n = 34). **P < 0.001, *P < 0.05 compared with the values at baseline.

**Figure 5:**
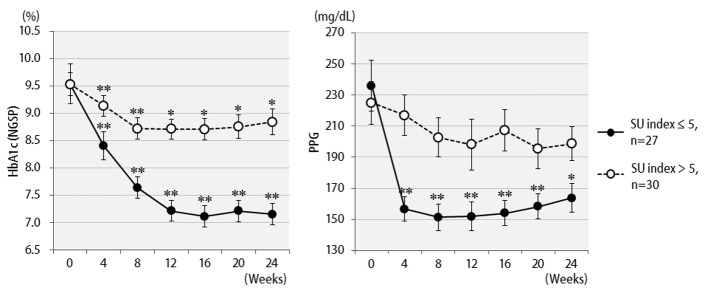
Changes in HbA1c (NGSP), 2 h postprandial plasma glucose for 24 weeks after liraglutide introduction by difference in SU index (glimepiride dose in mg × number of years of administration): over 5 (n = 27) or less than 5 (n = 30). **P < 0.001, *P < 0.05 compared with the values at baseline.

The efficacy of liraglutide in patients by BMI is shown in Figure 3. Contrary to initial expectations, the greatest efficacy (2.5% decrease in HbA1c levels) was observed in non-obese patients with BMI < 25 kg/m^2^. Efficacy decreased as the degree of obesity increased to a BMI of 25 kg/m^2^ or greater, and even further with a BMI of 30 kg/m^2^ or greater. Liraglutide is generally effective in markedly obese patients. However, the results of the present study revealed that this agent is not effective in patients with severe obesity. Efficacy according to the duration of type 2 diabetes is shown in Figure 4. Efficacy was clearly higher in patients who had a short duration of diabetes, i.e. less than 10 years, as compared to those with durations exceeding 10 years. In addition, history of SU drug use was examined using the SU index, which was defined as the dose in milligrams converted to an equivalent regimen of glimepiride multiplied by the number of years it was administered. The results of the differential analysis performed to ascertain whether SU index scores were lower or higher than 5 are shown in Figure 5. In the group that clearly had low SU index scores, liraglutide was observed to be markedly effective.

## Discussion

Incretins, which are gastrointestinal tract hormones, stimulate glucose-dependent insulin secretion [23]. Apart from the effects of incretins on the pancreas, GIP has been shown to cause accumulation of nutrients in adipocytes and calcium in bones through improvements in osteoblastic function, whereas GLP-1 exerts the action of inhibiting glucagon secretion in pancreatic α cells, promoting appetite suppression through the central nervous system, decreasing gastric emptying and providing cardio-protective effects [24]. When treated with incretin-associated agents, the pancreatic and extra-pancreatic effects of these agents can be anticipated in patients with type 2 diabetes, leading to favorable blood glucose control without increasing body weights [25-27]. When the treatment regimen involves GLP-1 analogue injection, however, serum concentrations of GLP-1 can reach non-physiological high levels [28], such that extra-pancreatic effects of GLP-1 that are not observed in patients who receive DPP-4 inhibitors can be expected.

In general, LEAD studies have demonstrated the efficacy and safety of liraglutide [8-13], because not only the amelioration of blood glucose elevations by liraglutide but also various extra-pancreatic benefits, such as decreases in body weight and systolic blood pressure, as well as amelioration of pancreatic β cell function and cardiovascular markers, have been demonstrated. The degree of obesity is higher in western than in Asian countries including Japan. In western countries, there are more patients with type 2 diabetes who exhibit greater insulin resistance. Thus, not only stimulation of insulin secretion but also extra-pancreatic effects, such as the action decreasing body weight following appetite control, can be expected with liraglutide treatment. Herein, we found that with regard to appetite suppression and associated decreases in body weight, the 0.9 mg dose of liraglutide was not effective in obese Japanese patients. These observations suggest that the mechanisms underlying the amelioration of blood glucose elevation by liraglutide treatment may differ between western and Japanese type 2 diabetes patients. As described above, many novel findings concerning the generally accepted efficacy of liraglutide to date were confirmed in the present study. In Japanese patients, 1) a 0.6 mg liraglutide dose was adequately effective in many patients. The effects of increasing the dose to 0.9 mg were limited in patients in whom 0.6 mg liraglutide had not been effective. 2) Efficacy was highest in patients who had comparatively low BMI (< 25 kg/m^2^). 3) SU drug use (SU index) as well as the duration of type 2 diabetes had a major influence on efficacy.

In recent years, GLP-1 receptor agonists have been classified as either long- or short-acting agents [29, 30]. While a high serum GLP-1 level is known to suppress the parasympathetic nervous system and decrease gastric emptying [31], sustained venous administration of GLP-1 reportedly weakens this response to delay gastric emptying [32]. This phenomenon might be attributable to the occurrence of tachyphylaxis in response to long-term maintenance of high GLP-1 concentrations. On the other hand, recent studies have focused on the appetite suppressing effects of liraglutide exerted via the ventromedial nucleus of the hypothalamus [33, 34]. Taken together, these observations suggest that the maximum allowed dose of 0.9 mg is insufficient for controlling appetite. The non-physiological elevation of GLP-1, to very high levels, during fasting also promotes insulin secretion. Thus, the insulin secretion-promoting effects of liraglutide while fasting may have an undesirable effect from the viewpoint of weight control.

Our findings indicate that at least in Japan, where the maximum dose of liraglutide covered by insurance is 0.9 mg, the efficacy of this drug for appetite suppression and associated weight loss in obese patients should not be expected. Rather, this agent is likely to be highly effective in non-obese patients. A low dose, just 0.6 mg of liraglutide, may be sufficient to promote insulin secretion in Japanese patients who have impaired insulin secretion. In fact, undesirable rebounds in body weight and blood glucose levels were observed in some patients with BMI of 35 kg/m^2^ or more after a 6-month course of treatment, whereas favorable blood glucose levels were maintained in non-obese patients after 6 months of continued treatment at the 0.6 mg dose. In addition, in order to effectively utilize this agent, liraglutide should be administered to patients who are in the early stages of diabetes, particularly before starting the administration of SU drugs. We herein obtained novel findings regarding the effective use of liraglutide.
